# Apology

**Published:** 1892-04

**Authors:** 


### EDITORIAL.

### Apology.

We regret extremely the delay in this issue of the Journal, which was caused by unavoidable matters. The first of a series of articles upon the various races of animals was unexpectedly stopped by failure to receive the necessary illustrations, and just at that time the managing editor was prevented for some days from attending to any desk work. We beg the lenient courtesy of our readers and promise additional matter in our next numbers to compensate for the shortcomings of the present one. We wish also to share our burden with the principals of the various veterinary schools which have just held their commencements, not one of whom has considered the graduation of his students of sufficient importance to the veterinary profession to send an account of it to us, in other form than in crude programs and inaccurate newspaper cuttings, so that we have been obliged to write for further details. In the next number we will publish an article upon the characteristic points of the various breeds of dogs, with illustrations, as decided by the judging at the recent Westminster Kennel Club's exhibition. Later we will publish similar articles upon horses, cattle and other animals following the large exhibitions of these animals. We beg to call attention to the advertisement of the U. S. Horse and Cattle Show Society, whose prize list will be of interest to the veterinarian, and can be had by addressing the Secretary.

